# 80. SARS-CoV-2 infection is associated with decreased reported physical fitness in a US military longitudinal cohort

**DOI:** 10.1093/ofid/ofac492.005

**Published:** 2022-12-15

**Authors:** Stephanie A Richard, Ann Scher, Jennifer Rusiecki, Celia Byrne, Catherine M Berjohn, Anthony C Fries, Tahaniyat Lalani, Alfred G Smith, Rupal Mody, Anuradha Ganesan, Nikhil Huprikar, Rhonda E Colombo, Christopher Colombo, David A Lindholm, Katrin Mende, Milissa Jones, Derek Larson, Evan C Ewers, Samantha Bazan, Charlotte Lanteri, David Saunders, Ryan C Maves, Jeffrey Livezey, Margaret Sanchez Edwards, Julia S Rozman, David R Tribble, Brian Agan, Simon Pollett, Mark P Simons, Timothy Burgess

**Affiliations:** Infectious Disease Clinical Research Program, Department of Preventive Medicine and Biostatistics, Uniformed Services University of the Health Sciences, Bethesda, MD, USA, Bethesda, MD; Uniformed Services University of the Health Sciences, Bethesda, Maryland; Uniformed Services University of the Health Sciences, Bethesda, Maryland; Uniformed Services University of the Health Sciences, Bethesda, Maryland; Naval Medical Center San Diego Division of Infectious Diseases, Infectious Disease Clinical Research Program, San Diego, CA; U.S. Air Force School of Aerospace Medicine, Dayton, Ohio; Naval Medical Center Portsmouth, Portsmouth, Virginia; Division of Infectious Diseases, Naval Medical Center, Portsmouth, Virginia, USA, Portsmouth, Virginia; William Beaumont Army Medical Center, El Paso, Texas; Infectious Disease Clinical Research Program, Department of Preventive Medicine and Biostatistics, Uniformed Services University of the Health Sciences, Bethesda, MD, USA, Walter Reed National Military Medical Center, Bethesda, Maryland; Walter Reed National Military Medical Center, Bethesda, Maryland; Infectious Disease Clinical Research Program, Department of Preventive Medicine and Biostatistics, Uniformed Services University of the Health Sciences, Bethesda, MD, USA, The Henry M. Jackson Foundation for the Advancement of Military Medicine, Madigan Army Medical Center Division of Infectious Diseases, Tacoma, Washington; Madigan Army Medical Center, Tacoma, Washington; Brooke Army Medical Center, Bethesda, Maryland; Infectious Disease Clinical Research Program, Department of Preventive Medicine and Biostatistics, Uniformed Services University of the Health Sciences, Bethesda, MD, USA, Bethesda, MD; Tripler Army Medical Center, Honolulu, Hawaii; Naval Medical Center San Diego, san diego, California; Fort Belvoir Community Hospital, Fort Belvoir, Virginia; Carl R. Darnall Army Medical Center, Fort Hood, Texas; Infectious Disease Clinical Research Program, Department of Preventive Medicine and Biostatistics, Uniformed Services University of the Health Sciences, Bethesda, Maryland; Uniformed Services University of the Health Sciences, Bethesda, MD, USA, Bethesda, Maryland; Wake Forest University School of Medicine, Winston-Salem, North Carolina; Uniformed Services University of the Health Sciences, Bethesda, MD, USA, Bethesda, Maryland; Infectious Disease Clinical Research Program, Department of Preventive Medicine and Biostatistics, Uniformed Services University of the Health SciencesHenry M. Jackson Foundation for the Advancement of Military Medicine, Bethesda, Maryland; Infectious Disease Clinical Research Program, Department of Preventive Medicine and Biostatistics, Uniformed Services University of the Health Sciences, Bethesda, MD, USA, Bethesda, MD; Uniformed Services University of the Health Sciences, Bethesda, Maryland; Infectious Disease Clinical Research Program, Department of Preventive Medicine and Biostatistics, Uniformed Services University of the Health Sciences, Bethesda, MD, USA, Bethesda, MD; Infectious Disease Clinical Research Program, Department of Preventive Medicine and Biostatistics, Uniformed Services University of the Health Sciences, Bethesda, MD, USA, Bethesda, MD; Infectious Disease Clinical Research Program, Department of Preventive Medicine and Biostatistics, Uniformed Services University of the Health Sciences, Bethesda, MD, USA, Bethesda, MD; Infectious Disease Clinical Research Program, Department of Preventive Medicine and Biostatistics, Uniformed Services University of the Health Sciences, Bethesda, MD, USA, Bethesda, MD

## Abstract

**Background:**

COVID-19 may have deleterious effects on the fitness of active duty US military service members. We seek to understand the long-term functional consequences of SARS-CoV-2 infection in this critical population, and in other military healthcare beneficiaries.

**Methods:**

The Epidemiology, Immunology, and Clinical Characteristics of Emerging Infectious Diseases with Pandemic Potential (EPICC) study is a longitudinal cohort study to describe the outcomes of SARS-CoV-2 infection in US Military Health System beneficiaries. Subjects provided information about difficulties experienced with daily activities, exercise, and physical fitness performance via electronic surveys. Subjects completed surveys at enrollment and at 1, 3, 6, 9, and 12 months.

**Results:**

5,910 subjects completed survey fitness questions, 3,244 (55%) of whom tested SARS-CoV-2 positive at least once during the period of observation. Over 75% of subjects were young adults and over half were male (Table 1). 1,093 (34.3%) of SARS-CoV-2-positive subjects reported new or increased difficulty exercising compared to 393 (14.8%) SARS-CoV-2 negative subjects (p < 0.01) (Table 2). The most commonly reported symptoms related to problems with exercise and activities were dyspnea and fatigue. Among the active-duty members who answered the question about their service-mandated physical fitness test scores, 43.2% of SARS-CoV-2-positive participants reported that their scores had worsened in the study period, compared with 24.3% of SARS-CoV-2 negative participants. Among SARS-CoV-2-positive subjects, reports of difficulty exercising and performing daily activities were highest within one month of the first positive test, decreasing in prevalence among the cohort only slightly to 24% and 18%, respectively, at 12 months (Figure 1).

**Conclusion:**

A substantial proportion of military service-members in this cohort have reported impairment of their service-mandated physical fitness scores after COVID-19; this proportion is significantly higher than those who are SARS-CoV-2 negative and persists to 12 months in many; similar complaints were reported among non-active duty. Further objective evaluation of post-COVID fitness impairment in this population is warranted.

**Disclosures:**

**Ryan C. Maves, MD**, AiCuris: Grant/Research Support|Sound Pharmaceuticals: Grant/Research Support|Trauma Insights, LLC: Advisor/Consultant **Julia S. Rozman, n/a**, Astra Zeneca: The HJF, in support of the USU IDCRP, was funded to conduct or augment unrelated Phase III Mab and vaccine trials as part of US Govt. COVID19 response **David R. Tribble, DrPH**, AstraZeneca: The HJF, in support of the USU IDCRP, was funded to conduct or augment unrelated Phase III Mab and vaccine trials as part of US Govt. COVID19 response **Simon Pollett, MBBS**, Astra Zeneca: The HJF, in support of the USU IDCRP, was funded to conduct or augment unrelated Phase III Mab and vaccine trials as part of US Govt. COVID19 response **Mark P. Simons, PhD**, AstraZeneca: The HJF, in support of the USU IDCRP, was funded to conduct or augment unrelated Phase III Mab and vaccine trials as part of US Govt. COVID19 response **Timothy Burgess, MD, MPH**, AstraZeneca: The HJF, in support of the USU IDCRP, was funded to conduct or augment unrelated Phase III Mab and vaccine trials as part of US Govt. COVID19 response.

